
JOURNAL INFORMATION
==============================
NLM Title Abbreviation: ScientificWorldJournal
Journal ID: ScientificWorldJournal
NLM Journal ID: 101131163
Journal ID: TSWJ

The Scientific World Journal

ISSN: 2356-6140
EISSN: 1537-744X
Publisher: Wiley

ARTICLE INFORMATION
==============================
PMCID: PMC6083965
PMID: 30147593
DOI: 10.1100/tsw.2001.123
Article ID: 170685
Article version: 1
Subjects: Short Report

Ascorbic Acid Augments Arsenic-Mediated Cell Death in Multiple Myeloma (MM) Cells

Grad Jennifer M. 1 2 *
Bahlis Nizar J. 2
Boise Lawrence H. 1 2
1 Department of Microbiology and Immunology, University of Miami School of Medicine, P.O. Box 016960 (R-138), Miami, FL, 33101, USA
2 Sylvester Cancer Center, University of Miami School of Medicine, P.O. Box 016960 (R-138), Miami, FL, 33101, USA
*Corresponding author.
Publication date: 2001
Electronic publication date: 2001 Nov 9
Volume: 1
First page: 110
Last page: 110
Copyright: Copyright © 2001 Jennifer M. Grad et al.
Copyright year: 2001
License: This is an open access article distributed under the Creative Commons Attribution License, which permits unrestricted use, distribution, and reproduction in any medium, provided the original work is properly cited.
License URL: https://creativecommons.org/licenses/by/3.0/

==============================
Miami *Nature Biotechnology* Short Reports
*TheScientificWorld* (2001) 1(S3), 110SR
ISSN 1532-2246; DOI 10.1100/TSW.2001.123

### ASCORBIC ACID AUGMENTS ARSENIC-MEDIATED CELL DEATH IN MULTIPLE MYELOMA (MM) CELLS

Jennifer M. Grad[1,2,]*, Nizar J. Bahlis[2], and Lawrence H. Boise[1,2]

Department of Microbiology and Immunology[1], Sylvester Cancer Center[2], University of Miami School of Medicine, P.O. Box 016960 (R-138), Miami, FL, 33101 USA
* Corresponding author.

**INTRODUCTION.** Greater than 90% of MM patients progress to a chemoresistant state (1). New therapies are clearly needed for the treatment of refractory MM that overcome or bypass the adaptive cellular changes associated with refractory disease: P-glycoprotein expression, increased Bcl-$x_L$ expression, elevated glutathione (GSH). Arsenic cytotoxicity is independent of Bcl-$x_L$ expression (2), making it a promising anti-neoplastic agent for MM. Ascorbic acid (AA) has recently been shown to deplete GSH via autooxidation and subsequent reduction reactions (3). Therefore, we hypothesized that the combination of arsenic and AA is likely to induce apoptosis in refractory MM cells.

**METHODS.** Cell viability was measured by Annexin-FITC and PI staining and FACScan flow cytometry. Intracellular GSH was measured using the Glutathione Assay Kit (CalBiochem). $H_2O_2$ and $O^{-2}$ production were measured by flow cytometry using $H_2$DCFDA and hydroethidine (Molecular Probes), respectively. Mitochondrial membrane potential ($\Delta\Psi_m$) was measured by flow cytometry using TMRE (Molecular Probes). Bone marrow (BM) aspirates were obtained from consenting MM patients and mononuclear cells isolated by ficoll purification. After 48h treatment, cells were triple stained with a mouse anti-human CD38-PE antibody, a mouse anti-human CD45-CyChrome antibody, and Annexin-FITC.

**RESULTS AND DISCUSSION.** All three MM cell lines we tested, 8226/S, 8226/Dox40 (PgP+), U266, were sensitive to arsenic in a dose-dependent fashion. U266 cells stably expressing exogenous Bcl-$x_L$ were initially resistant to arsenic (2 μM), but less than 35% viable after 96h of treatment. Since AA is well tolerated *in vivo*, and has been shown to deplete GSH (3), a critical determinant of arsenic sensitivity (4), we tested the ability of AA (100 μM) to decrease GSH and potentiate arsenic cytotoxicity in MM cell lines. AA depleted GSH without affecting cell viability and the combination of AA and arsenic significantly increased apoptosis in all four cell lines. Similarly, AA alone had no effect on $O^{-2}$ production, but the combination of AA and arsenic resulted in higher levels of $O^{-2}$ than with arsenic alone. A similar pattern was observed when changes in $\Delta\Psi_m$ were measured, consistent with arsenic exerting cellular cytotoxic effects via the generation of ROS, resulting in free radical cellular damage. Importantly, arsenic induced apoptosis (26.8%±3.9) in malignant plasma cells ($CD38^+CD45^-$) from MM patients, while normal BM cells ($CD38^-$) were relatively unaffected (7.2% ± 1.9). The combination of AA and arsenic was significantly more cytotoxic than etoposide in the $CD38^+CD45^-$ population ($p<0.002$). However, viabilities of $CD38^-$ cells treated with arsenic, AA+arsenic, or etoposide were not significantly different. AA treatment alone has the potential to be a safe and effective chemosensitizing agent in an arsenic-based therapy. These pre-clinical studies have led to an NCI-sponsored Phase I/II trial (University of Miami) to assess the combination of arsenic and AA as a potential therapy for plateau and refractory MM patients.

**REFERENCES.**

1. Hallek, M., Bergasagel, P.L., and Anderson, K. (1998) Blood 91(1), 3-21
2. Perkins, C., Kim, C.N., Fang, G., and Bhalla, K.N. Blood 95(3), 1014-1022
3. Dai, J., Weinberg, R.S., Waxman, S., and Jing, Y. (1999) Blood 93(1), 268-277
4. Yang, C.H., Kuo, M.L., Chen, J.C., and Chen, Y.C. Br. J. Cancer 81(5), 796-799
